# Low-dose oxytocin delivered intranasally with Breath Powered device affects social-cognitive behavior: a randomized four-way crossover trial with nasal cavity dimension assessment

**DOI:** 10.1038/tp.2015.93

**Published:** 2015-07-14

**Authors:** D S Quintana, L T Westlye, Ø G Rustan, N Tesli, C L Poppy, H Smevik, M Tesli, M Røine, R A Mahmoud, K T Smerud, P G Djupesland, O A Andreassen

**Affiliations:** 1 NORMENT, KG Jebsen Centre for Psychosis Research, Division of Mental Health and Addiction, Oslo University Hospital, University of Oslo, Oslo, Norway; 2 Department of Psychology, University of Oslo, Oslo, Norway; 3 Smerud Medical Research International AS, Oslo, Norway; 4 OptiNose US Inc, Yardley, PA, USA; 5 OptiNose AS, Oslo, Norway

## Abstract

Despite the promise of intranasal oxytocin (OT) for modulating social behavior, recent work has provided mixed results. This may relate to suboptimal drug deposition achieved with conventional nasal sprays, inter-individual differences in nasal physiology and a poor understanding of how intranasal OT is delivered to the brain in humans. Delivering OT using a novel ‘Breath Powered' nasal device previously shown to enhance deposition in intranasal sites targeted for nose-to-brain transport, we evaluated dose-dependent effects on social cognition, compared response with intravenous (IV) administration of OT, and assessed nasal cavity dimensions using acoustic rhinometry. We adopted a randomized, double-blind, double-dummy, crossover design, with 16 healthy male adults completing four single-dose treatments (intranasal 8 IU (international units) or 24 IU OT, 1 IU OT IV and placebo). The primary outcome was social cognition measured by emotional ratings of facial images. Secondary outcomes included the pharmacokinetics of OT, vasopressin and cortisol in blood and the association between nasal cavity dimensions and emotional ratings. Despite the fact that all the treatments produced similar plasma OT increases compared with placebo, there was a main effect of treatment on anger ratings of emotionally ambiguous faces. Pairwise comparisons revealed decreased ratings after 8 IU OT in comparison to both placebo and 24 IU OT. In addition, there was an inverse relationship between nasal valve dimensions and anger ratings of ambiguous faces after 8-IU OT treatment. These findings provide support for a direct nose-to-brain effect, independent of blood absorption, of low-dose OT delivered from a Breath Powered device.

## Introduction

A growing body of evidence demonstrates a critical role of oxytocin (OT) in social cognition and behavior.^1, 2, 3^ For instance, a single administration of OT increases empathy,^4, 5^ trust,^6^ group-serving behaviors,^7, 8^ sensitivity of eye gaze^9^ and theory-of-mind performance in healthy individuals^10^ and in patients with psychiatric disorders.^11^ Due to this burgeoning literature, OT has been proposed as a novel therapy for disorders characterized by social dysfunction, such as autism and schizophrenia spectrum disorders.^12, 13^ In spite of initial promise, however, recent work has either failed to identify changes in social behavior after OT administration^14^ or has provided results that are only significant in specific subgroups or contexts.^15^ Although these mixed results have been largely attributed to such contextual and individual differences,^16^ factors that may influence biological activity of intranasal exogenous OT—such as dose, nasal cavity dimensions and delivery method—have yet to be thoroughly investigated.^15, 17, 18^

The two-level model of OT response highlights the important role of targeted intranasal delivery and nasal physiology in intranasal OT administration, describing three pathways to the central nervous system (CNS) from the nose.^18^ Intranasally administered OT can enter the CNS indirectly via mechanisms such as capillary uptake in the nasal vasculature with subsequent penetration of the blood–brain barrier (BBB) in the walls of the cerebral capillaries or through the more leaky barrier between the blood and the cerebrospinal fluid in the choroid plexus (that is, blood-to-cerebrospinal fluid barrier).^15, 17, 19, 20, 21^ However, only very small—but perhaps still biologically significant^19^—amounts of OT enter the CNS via these indirect routes.^22^ Alternatively, perineural bulk transport along ensheathed channels surrounding the olfactory and trigeminal nerve fiber pathways offer two rapid and direct routes from the nasal cavity to the CNS, circumventing the BBB.^15, 18, 23^ Olfactory nerve fibers innervate a limited segment of the deep upper narrow nasal passage, while the trigeminal nerve provides sensory and parasympathetic innervation to the deep upper and posterior segments of the nasal cavity. To reach these crucial targets for nose-to-brain delivery, intranasally administered OT needs to initially overcome, among other challenges, the problem of delivery to areas beyond the nasal valve.^15^ This valve region is a narrow triangular-shaped opening located 2–3 cm from the nostrils.^24^ Given the barrier that this dynamic structure presents, it is unsurprising that traditional nasal delivery devices deposit mainly in or anterior to this narrow valve with a minimal fraction going on to reach the upper posterior segments housing the key nose-to-brain target sites.^25, 26, 27^ Nasal valve dimensions vary between individuals and over time, and are dependent on multiple factors, including overall health,^28^ septal deviation,^29^ mucosal inflammation and nasal polyps.^30^ However, despite the important role of the nasal valve in intranasal drug administration it has yet to be considered in the context of OT delivery. Nasal valve dimensions could provide a measurable parameter that could further inform the role of delivery mechanism and deposition pattern on treatment efficacy.

Improvement of intranasal delivery of OT to target sites beyond the nasal valve may yield improved pharmacodynamic (PD) effects. The recent development of Breath Powered closed-palate Bi-Directional technology (Supplementary Figure S1; OptiNose, Oslo, Norway) creates an opportunity to investigate a new form of intranasal delivery that is hypothesized, by virtue of direct nose-to-brain activity, to produce PD effects in the brain disproportionate to what would be achieved by absorption into the blood and transport across the BBB into the brain. It is reasonable to surmise that this type of targeted delivery may improve the reliability, therapeutic index and magnitude of OT treatment effects due to improved drug deposition;^15, 31, 32^ however, no prior research has investigated the PD response to OT delivered using this device.

Though generally accepted, the assumption that nasal delivery is an effective way of delivering OT to modulate social cognition and behavior has not been experimentally scrutinized in humans.^15^ Early work demonstrated that intravenous (IV) administration can influence social behavior and cognition^33, 34^—presumably via blood absorption and subsequent action across the BBB—however, all subsequent human studies assessing the effect of OT on cognitive functions have used methods that deliver OT via the nasal cavity. Although there is a strong theoretical basis that intranasal delivery is a more appropriate means of administering OT to the CNS given increases in cerebrospinal fluid concentrations of OT after intranasal administration,^35^ a controlled comparison of PD effects after intranasal (that is, nose-to-brain) and IV (that is, transportation across the BBB) administration is of interest.^18^ Greater central effects (for example, social cognition) after targeted intranasal drug delivery, in the presence of comparable blood exposure, would provide much-needed support for direct nose-to-brain activity.

Another hurdle for the development of OT as a therapeutic intervention is the definition of an optimal dosing regimen. The majority of intranasal OT studies evaluated between 20 and 40 international units (IU).^36^ However, there is no comprehensive empirical evidence substantiating this dosage, despite calls for such research^18, 37, 38^ and successful efforts in other disciplines (for example, obstetrics^39^). The negative long-term effects of OT treatment observed in non-human adolescent mammals,^40^ and the presence of OT and cross-reactive vasopressin (AVP) receptors throughout the body^41^ that are involved in a variety of homeostatic functions related to observed side effects,^42^ further reinforces the axiomatic importance of identifying the lowest effective doses of OT.

To date, no controlled clinical trial has investigated the effect of different intranasal OT dosages vs IV OT on social cognition or the role of nasal dimensions in OT treatment response. This randomized, double-blind, double-dummy, four-way crossover trial in healthy volunteers compared PD outcome on social cognition, as indexed by performance on a computerized emotional faces rating paradigm, between four treatments: ‘low dose' (8 IU) OT delivered with the Breath Powered OptiNose device (OPN-OT), ‘higher dose' (24 IU) OPN-OT, OT delivered intravenously (IV OT; 1 IU) and placebo. Although the comparison of OT administration with a traditional hand-actuated spray pump is of theoretical interest, this would require the inclusion of additional OT and placebo arms and an especially complex double-dummy design for appropriate double-blinding (that is, successive administration of solution with the OPN-OT device and hand-actuated device, with associated concerns regarding a ‘washout' effect). Moreover, doubling the delivered volume from 300 μl per nostril would by far exceed the recommended maximum volume and increase drug drip-out and swallowing. Primary outcomes were the evaluation of facial emotional expression and secondary outcomes including pharmacokinetic profiles and ratings of trustworthiness. First, we hypothesized a main effect of OPN-OT on the perceived intensity of anger, given neural,^43^ behavioral^44, 45, 46, 47^ and anxiolytic^48^ evidence for the role of OT in processing social threat stimuli, and that this effect would be more pronounced with ambiguous emotional stimuli compared with stimuli with less ambiguous emotional expressions in light of prior research^43^ and the general ambiguity of social signals.^48^ Although OT appears to influence the processing of negative social stimuli in general, this effect may be stronger in response to anger than fear stimuli.^46^ Second, we examined the dose-dependency of these PD effects of OPN-OT. Last, we investigated the relationship between nasal valve cross-sectional area and evaluation of facial emotional expression along with impact of OT on trust ratings of the same facial stimuli. To characterize pharmacokinetics and evaluate potentially different relationships between pharmacokinetics and PD by method of drug delivery, we also explored the time course of blood plasma concentrations of OT and physiologically interacting substances AVP^49^ and cortisol,^50^ to index physiological stress via hypothalamus–pituitary–adrenal axis activity.^51^ Modulation, or disproportionate modulation, of social cognition (that is, of PD) after OPN-OT administration, but not after IV OT producing comparable blood exposure, would provide evidence that OPN-OT is, at least in part, directly acting on the brain via upper posterior nasal pathways rather than across the BBB.

## Materials and methods

### Participants

Participants were recruited among students at the University of Oslo through advertisements. Eligible participants were males in good physical and mental health between the ages of 18 to 35. Exclusion criteria included use of any medications within the last 14 days, history of alcohol or drug abuse, clinically relevant history of physical or psychiatric illness and intelligence quotient <75.

A screening visit occurred between 3 and 21 days before randomization. Trained graduate students administered the Wechsler Abbreviated Scale of Intelligence^52^ and the Mini-International Neuropsychiatric Interview^53^ to index intelligence quotient and confirm the absence of psychiatric illness, respectively. A physical examination was performed, including ECG and the collection of routine blood samples. As per recommendations,^15^ an otolaryngologist confirmed normal nasal anatomy and patency in participants and acoustic rhinometry data were collected (SRE 2000; Rhinometrics, Lynge, Denmark). Nasal valve dimensions (that is, the minimum cross-sectional area) and nasal cavity volume measures (TV0–5, total volume from nostril to 5 cm deep; TV2–5, total volume from 2 to 5 cm deep) were calculated from the acoustic rhinometry data.

### Experimental design

A randomized, placebo-controlled, double-blind, double-dummy, four-period crossover design was used. Participants were randomized to one of four treatment sequences, using a four-period four-treatment Latin square method (ACDB–BDCA–CBAD–DABC in a 4:4:4:4 ratio) with a period of at least 6 days between treatments to prevent potential carryover and/or practice effects. The study monitor (Smerud Medical Research International, Oslo, Norway) performed randomization and both the participants and research team were masked to treatment using visually matching intranasal devices and IV apparatus during data collection. A pragmatic approach was taken for sample size determination reflecting the difficulty of execution, complexity and burden on study subjects of the study. This trial was approved by the Regional Committee for Medical and Health Research Ethics (REC South East) and registered at http://clinicaltrials.gov (NCT01983514). Participants provided written informed consent and were reimbursed NOK 750 (approximately USD $125) per testing session.

### Breath Powered delivery device and OT administration

The Breath Powered, closed-palate, bi-directional nasal delivery device has a mouthpiece connected in series to a delivery unit and a sealing nosepiece optimized for nose-to-brain delivery (Supplementary Figure S1).^23, 32^ When the user slides the bespoke nosepiece into one nostril, it forms a seal with the nostril opening and mechanically expands the narrow slit-shaped part of the nasal valve. The intraoral pressure created by blowing into the mouthpiece elevates the soft palate and creates an airtight seal, which isolates the nasal cavity from the rest of the respiratory system, thereby reducing drug loss from swallowing. The pressure of the patient's exhaled breath is released when the patient actuates the spray pump, expanding the narrow nasal passages and propelling OT deeply past the nasal valve for improved drug deposition on target regions,^26^ after which the airflow balances pressure across the soft palate enabling the exhaled breath to travel in the opposite direction and exit out the other nostril.

The OT and placebo formulations were supplied by a cGMP manufacturer (Sigma-Tau Industrie Farmaceutiche Riunite, Rome, Italy) to a pharmaceutical service provider (Farma Holding, Oslo, Norway) for the filling of the drug and placebo formulations into the OPN-OT devices. The IV OT (10 IU/ml; Grindeks, Riga, Latvia) and placebo formulations (0.9% sodium chloride) were added to a 0.9% sodium chloride solution for infusion shortly before administration (600 mL/h over 20 min). The IV OT dosage and infusion rate was guided by a pilot study (described in Section 1 and Supplementary Figure S2 of Supplementary Information), which determined that that 1 IU delivered over 20 min generates peripheral OT concentrations equivalent to 24 IU delivered intranasally. All the participants self-administered an intranasal treatment using a Breath Powered device and also received an IV solution—either OT or placebo depending on randomization—in all treatment periods (solution ingredients and administration regimen described in Section 2 of Supplementary Information).

### Experimental testing session procedure

At the beginning of each experimental session, exclusion and inclusion criteria were confirmed and the State–Trait Anxiety Inventory^54^ was administered. Blood was sampled and acoustic rhinometry was performed on all the participants.

### Primary outcome parameters

Participants completed the social cognition task 40 min after treatment in a magnetic resonance imaging scanner while functional magnetic resonance imaging and physiology data were recorded (results to be reported separately). Participants were presented with visual stimuli through magnetic resonance imaging-compatible goggles (VisualSystem; NordicNeuroLab, Bergen, Norway) using E-Prime 2.0 (Psychology Software Tools, PA, USA) and responded using a grip response collection system (ResponseGrip, NordicNeuroLab).

For the primary emotional expression evaluation outcome measure, participants were presented with 20 male and 20 female faces (as used previously^55^) displaying angry, happy and emotionally ambiguous facial expressions (derived from the Karolinska Directed Emotional Faces database^56^) and 20 images of geometrical shapes (data not presented). Following each presentation, participants were asked either: *How angry is this person?* (anchors: *not angry*—*very angry*) or *How happy is this person?* (anchors: *not happy*—*very happy*). Q2 was always the same: *How much would you trust this person?* (anchors: *not at all*—*very much*). Participants were asked to rank their answer on a visual analog scale from 1 to 5, with location of the cursor on the visual analog scale randomized for each question. Mean ratings for each of the questions were averaged per session within each of the emotional categories, yielding seven behavioral variables (Q1: Happy face—happy, Happy face—angry, ambiguous face—happy, ambiguous face—angry, angry face—happy, angry face—angry; Q2: Trust). These stimuli and questions were chosen to assess three levels of emotion perception; ambiguous, non-ambiguous with corresponding cues and ratings (for example, angry ratings of angry faces) and non-ambiguous with conflicting cues and ratings (for example, angry ratings of happy faces).

### Pharmacokinetics

Blood samples were collected to assess peripheral levels of OT, AVP and cortisol at baseline and five time points after the completion of the 20-min IV administration (0, 10, 30, 60 and 120 mins) throughout the session. Up to two punctures with catheter placement were made to collect these blood samples, which were centrifuged at 4 °C within 5 min of blood draw with plasma frozen at −80 °C. Enzyme-linked immunosorbent assay using commercial kits (Enzo Life Sciences, Farmingdale, NY, USA) was performed using standard techniques (including sample extraction^57^).

### Safety measures

At various points (−20, 0, 10, 30, 60 and 120 mins relative to start of functional magnetic resonance imaging) throughout testing, participants reported the presence and severity of any adverse effects.

### Statistical analysis

Analysis was conducted using IBM SPSS Statistics version 22 (IBM, Armonk, NY, USA) to explore pharmacokinetics and examine the impact of treatment on outcome measures. A linear mixed-model (LMM) approach was adopted^58^ for the analysis of emotional expression evaluation, pharmacokinetics, state anxiety and trustworthiness. In contrast to a repeated-measures analysis of variance approach, LMM allows for the inclusion of participants in the analysis even if data are missing for some of the treatment conditions. All the models were fitted using an unstructured matrix. For any significant main effects (that is, *P*<0.05), *post hoc* tests were performed with the adjustment of critical *P*-values to correct for multiple comparisons using a 5% false discovery rate.^59^ Experimental treatment was both a fixed and repeated effect in the LMM testing the impact of treatment on emotion and trustworthiness ratings. To investigate the impact of treatment on anxiety and blood plasma concentration of OT, AVP and cortisol, an LMM was fitted with three fixed factors (treatment, time, treatment × time) and one repeated factor (treatment).

To investigate whether nasal environments changed between treatment conditions, a repeated-measures multivariate analysis of variance was performed with three dependent variables; minimum cross-sectional area, TV0–5 and TV2–5. As nasal valve dimensions may differ according to an individuals' overall size and age, Pearson correlation coefficients were also calculated to assess the relationship between these factors at the time of screening. The correlation between the posttreatment ratings of anger in emotionally ambiguous faces and nasal valve dimensions was then calculated. Bayes Factors using the Jeffreys–Zellner–Siow prior^60^ were calculated for these correlations to assess the strength of evidence for the null and alternative hypotheses. Confidence intervals (CIs) for the difference between correlations for each treatment condition were calculated using Zou's asymptotic method^61^ to compare the strength of correlation to investigate whether the relationship between nasal valve dimensions and anger ratings of ambiguous faces was significantly greater for some treatment conditions than others. As these variables are highly related due to measurements being taken from the same individual,^62^ the CIs were adjusted to account for overlap using the Fisher Z transformation.

## Results

Fifty-seven male volunteers were assessed for eligibility and 18 participants aged 20–30 years (M=23.81, s.d.=3.33) were randomized (Supplementary Figure S3). Two participants withdrew after enrollment (one withdrew after the first session (Placebo) and the other withdrew after completing three sessions (8 IU OPN-OT, IV OT, placebo)). Data from these participants are not included in the analyses.

### Emotional expression evaluation

**Figure 1:**
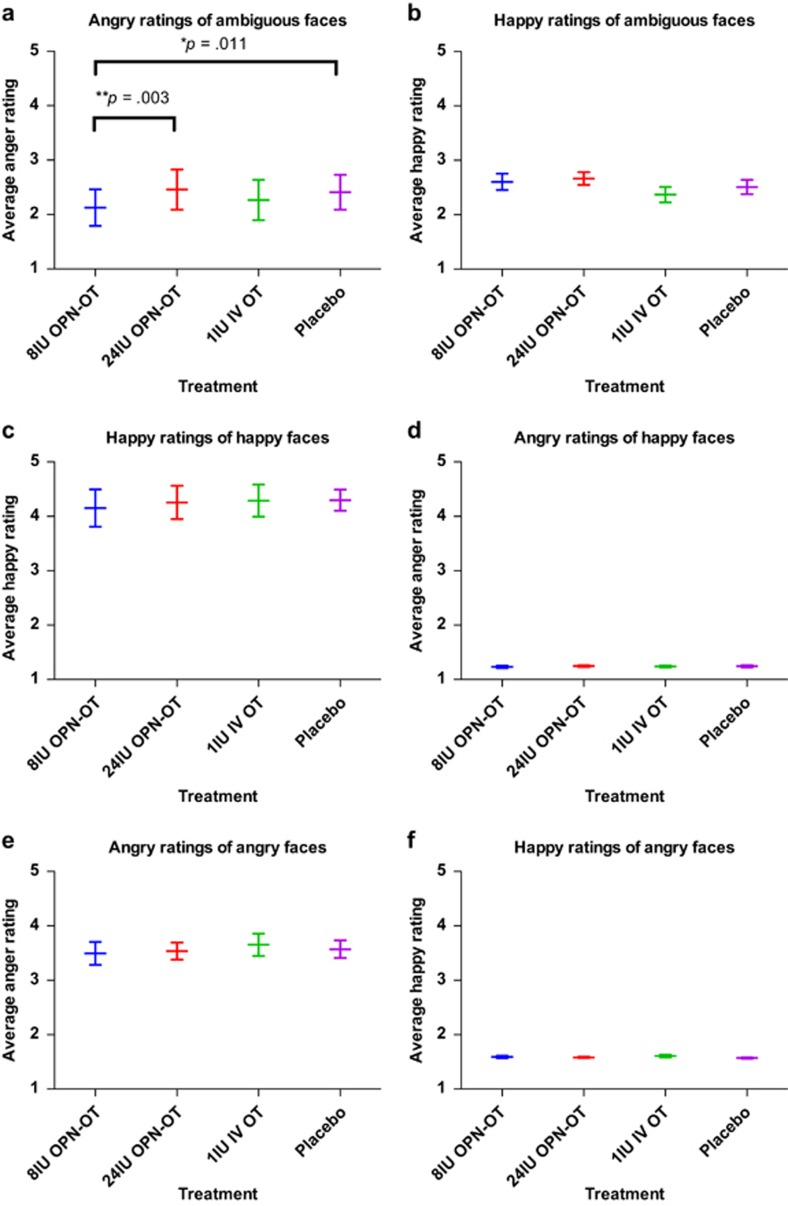
Mean emotional ratings by stimulus and treatment. Angry ratings of emotionally ambiguous faces were reduced after the administration of 8 IU OPN-OT in comparison with placebo and 24 IU OPN-OT (**a**; FDR correction applied, *q*=0.05, revised critical value of *P*<0.017 for *post hoc* comparisons). There were no main effects for any of the other evaluation categories (**b**–**f**). Emotion ratings can theoretically range from 1 to 5 and error bars represent standard error of the mean. *P<0.05. **P<0.01. FDR, false discovery rate; IU, international unit; IV, intravenous; OPN-OT, OT delivered with the Breath Powered OptiNose device; OT, oxytocin.

Table 1 and Figure 1 summarize the behavioral data. Due to equipment difficulties, data were not collected during two (out of 64) testing sessions. An LMM revealed a significant main effect of treatment in the ratings of anger when presented with ambiguous faces (F(3,14.72)=7.62, *P*=0.003; Figure 1a). Follow-up pairwise comparisons (*q*=0.05, revised critical value of *P*<0.017) indicated that angry ratings for ambiguous faces were significantly reduced in the 8 IU OPN-OT treatment condition in comparison with both the placebo (*P*=0.011; mean decrease=17%, SE decrease 6%) and 24 IU OPN-OT (*P*=0.003; mean decrease=17%, SE decrease 5%) treatments. There were no main effects of treatment observed for other emotional categories or trustworthiness ratings collapsed across emotional categories or for the anger or ambiguous faces. There was a main effect of treatment in trustworthiness ratings of the happy faces (F(3,14.67)=3.32, *P*=0.049) indicating different ratings of trustworthiness depending on experimental treatment, however, none of the follow-up pairwise comparisons survived false discovery rate correction (*q*=0.05, revised critical value of *P*<0.008).

**Figure 2:**
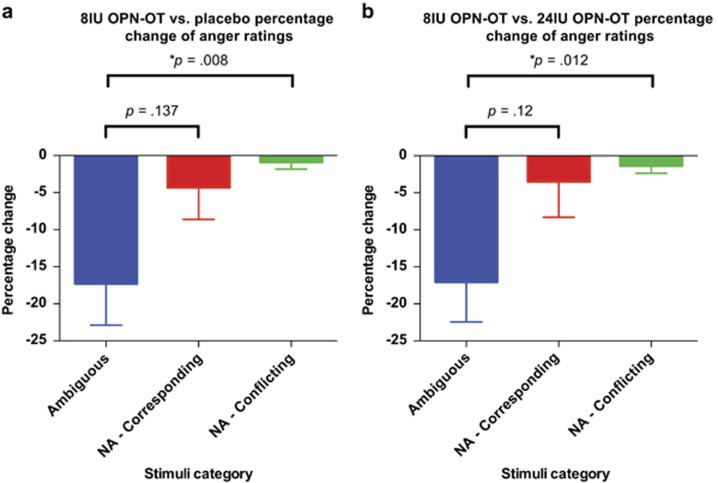
Percentage reduction in anger ratings was greater when presented with ambiguous faces compared with non-ambiguous faces (with corresponding or conflicting cues) after 8 IU OPN-OT compared with both placebo (**a**) and 24 IU OPN-OT (**b**) treatments (FDR correction applied, *q*=0.05, revised critical value of *P*<0.025 for *post hoc* comparisons). Ambiguous indicates anger ratings of ambiguous faces; NA-corresponding indicates anger ratings of non-ambiguous faces with corresponding cues; NA-conflicting indicates anger ratings of non-ambiguous faces with conflicting cues. Error bars represent s.e.m. *P<0.05. FDR, false discovery rate; IU, international unit; IV, intravenous; OPN-OT, OT delivered with the Breath Powered OptiNose device; OT, oxytocin.

To explore the specificity of the effect for ambiguous faces (vs non-ambiguous faces with corresponding cues and non-ambiguous with conflicting cues) a percentage change score was calculated comparing ratings after 8 IU OPN-OT and placebo treatments, and comparing 8 IU OPN-OT with 24 IU OPN-OT treatments (that is, the treatment comparisons that demonstrated significant differences in emotional ratings). Stimuli category was both a fixed and repeated effect in an LMM to assess the impact of stimuli category on the reduction of anger ratings. For the LMM comparing percentage change between the 8 IU OPN-OT and placebo treatment, there was a main effect for stimuli type (F(2,14.42)=4.79, *P*=0.025; Figure 2a). Follow-up pairwise comparisons to the ambiguous stimuli category (*q*=0.05, revised critical value of *P*<0.025) indicated that the percentage reduction of anger ratings of ambiguous stimuli was significantly reduced in comparison with the non-ambiguous/conflicting stimuli (*P*=0.012). For the LMM comparing percentage change between the 8 IU OPN-OT and 24 IU OPN-OT treatment, there was a main effect for stimuli type (F(2,14.05)=7.01, *P*=0.007; Figure 2b). Follow-up pairwise comparisons to the ambiguous stimuli category (*q*=0.05, revised critical value of *P*<0.025) indicated that the percentage reduction of anger ratings of ambiguous stimuli was significantly reduced in comparison with the non-ambiguous/conflicting stimuli (*P*=0.008).

### Pharmacokinetic data

Out of 384 possible data points, 12 OT, 26 AVP and 18 cortisol plasma concentration assessments were excluded due to technical issues relating to blood sample collection or analysis (for example, difficulty drawing blood, too little blood volume for analysis).

### Oxytocin blood plasma concentration

**Figure 3:**
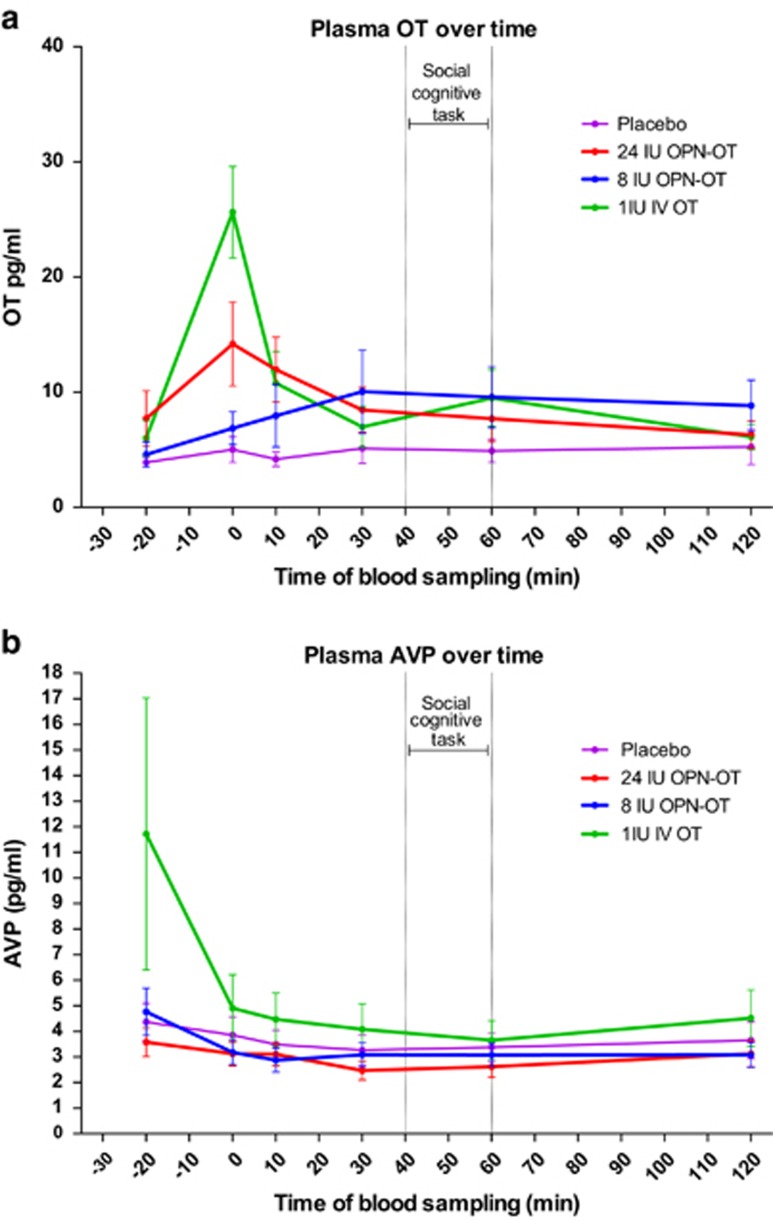
Pharmacokinetics of plasma OT (**a**) and AVP (**b**) after the administration of 8 IU OPN-OT, 24 IU OPN-OT, IV OT and placebo. Error bars represent s.e.m. IU, international unit; IV, intravenous; OPN-OT, OT delivered with the Breath Powered OptiNose device; OT, oxytocin.

The 4 (treatment) × 6 (time) LMM showed a significant main effect of treatment on OT blood plasma concentration (F(3,90.34)=12.42, *P*<0.001; Figure 3a), with pairwise comparisons (*q*=0.05, revised critical value of *P*<0.025) showing that plasma OT concentration was significantly increased in the IV (*P*<0.001), 8 IU OPN-OT (*P*<0.01) and 24 IU OPN-OT (*P*<0.001) treatments compared with placebo. Importantly, none of the pairwise comparisons between active treatment conditions reached significance. There was also a significant main effect for time (F(5,90.74)=5.81, *P*<0.001), with follow-up pairwise analyses (*q*=0.05, revised critical value of *P*<0.017) indicating significantly increased plasma OT immediately after IV solution administration in comparison with baseline (*P*<0.001) and all post-baseline time points (10, 30, 60 and 120 min). There was also a significant condition × time interaction, F(15,90.5)=2.67, *P*=0.002. Follow-up tests revealed a simple effect for time in the IV OT condition (F(5,87.15)=9.67, *P*<0.001), with significantly higher concentrations shortly after IV administration compared with all other time points (all *P*<0.001). There was no effect of condition on OT concentration just before (F(3,15.16)=1.28, *P*=0.32) or just after (F(3,15.02)=2.35, *P*=0.1) the completion of the social cognition task. An additional assessment of the main effect of condition on the percentage change of OT concentration compared with baseline at 30 min (F(3,15.26)=1.5, *P*=0.25) and 60 min (F(3,15.04)=0.63, *P*=0.61) after treatment revealed no significant differences (Supplementary Figure S4).

### Vasopressin blood plasma concentration

For the 4 (treatment) × 6 (time) LMM, there was a significant main effect of treatment on AVP blood plasma concentration (F(3,82.42)=4.55, *P*=0.005; Figure 3b). Follow-up pairwise comparisons (*q*=0.05, revised critical value of *P*<0.0083) revealed plasma AVP concentration was significantly decreased after 24 IU OPN-OT treatment in comparison with placebo (*P*=0.008) and IV OT (*P*=0.013), and significantly decreased after 8 IU OPN-OT treatment in comparison with IV OT (*P*=0.023). There was no significant main effect of time (F(5,90.63)=1.81, *P*=0.12) or treatment × time interaction, F(15,82.46)=1.03, *P*=0.434.

### Cortisol blood plasma concentration

For the 4 (treatment) × 6 (time) LMM, there was a significant main effect of treatment on cortisol blood plasma concentration (F(3,84.77)=4.82, *P*=0.004; Supplementary Figure S5). Follow-up pairwise comparisons (*q*<0.05, revised critical value of *P*<0.017) revealed significantly increased cortisol concentration following 1 IU IV OT treatment compared with placebo (*P*=0.01) and 24 IU OPN-OT (*P*<0.001), but not 8 IU OPN-OT. There was a significant main effect of time on cortisol blood plasma concentration (F(5,90.07)=2.4, *P*=0.04), but no significant follow-up pairwise comparisons were found. Finally, there was no significant treatment × time interaction (F(15,84.72)=0.421, *P*=0.969).

### State anxiety and adverse events

For the 4 (treatment) × 2 (time) LMM, there were no main effects of treatment (F(3,30)=0.27, *P*=0.84) or time (F(1,30)=0.18, *P*=0.67) or the treatment × time interaction on ratings of state anxiety (F(3,30)=0.98, *P*=0.42). Adverse events (for example, brief dizziness) were distributed across all four treatments (8 IU OPN-OT, three reports; 24 IU OPN-OT, two reports; IV OT, three reports; placebo, two reports).

### Nasal valve dimensions

Repeated measures multivariate analysis of variance indicated no main effect of treatment condition for nasal cavity dimensions (F(9,104.8)=0.41, *P*=0.93). There was no relationship between age (*r*=0.06, 95% CI (−0.45, 0.54), *n*=16, *P*=0.84) and body mass index (*r*=−0.07, 95% CI (−0.55, 0.44), *n*=15, *P*=0.015) with nasal valve dimensions at the time of screening.

**Figure 4:**
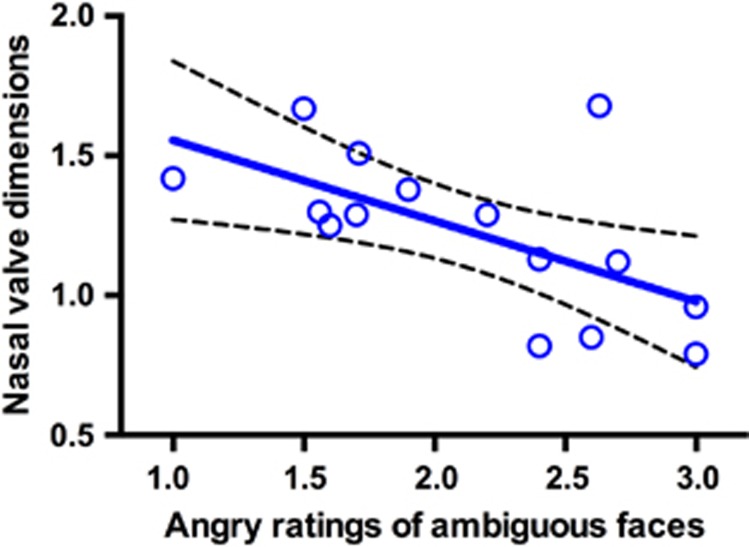
The relationship between angry ratings of ambiguous faces after 8 IU OT treatment and bilaterally summed mean cross-sectional areas in cm^2^ of the nasal valves with best-fit line and 95% confidence band. The significant inverse relationship indicates that individuals with wider nasal valves rate ambiguous faces as less angry after 8 IU OPN-OT administration.

Analysis revealed a significant relationship between nasal valve dimensions (summed minimum cross-sectional area) and the anger ratings of neutral faces after 8 IU OPN-OT treatment (*r*=−0.61, 95% CI (−0.85, −0.14), *n*=15, *P*=0.015; Figure 4) with a corresponding Bayes factor (*B*) of 3.62, representing substantial evidence that these two variables are related.^63^ There was no relationship between nasal valve dimensions and anger ratings of ambiguous faces after 24 IU OT (*r*=−0.14, 95% CI (−0.59, 0.38), *n*=16, *P*=0.6; *B*=0.22), IV OT (*r*=0.11, 95% CI (−0.43, 0.59), *n*=15, *P*=0.7; *B*=0.21) or placebo (*r*=0.04, 95% CI (−0.46, 0.53), *n*=16, *P*=0.88; *B*=0.19), with all respective Bayes factors providing evidence that these variables were not related. A comparison of the correlation coefficients^61^ also revealed a significant difference between the correlations of the 8 IU and placebo, (*r*=−0.65 (−1.1, −0.06)) and IV conditions (*r*=−0.72 (−1.4, −0.2)), but no significant difference in the correlation with 24 IU treatment (*r*=−0.42 (−0.97, 0.06)).

## Discussion

In this double-blind, placebo-controlled crossover trial in healthy volunteers, we have demonstrated that 8 IU OPN-OT treatment reduces the perception of anger in emotionally ambiguous facial stimuli. Importantly, the current findings are the first to suggest that a low dose of OT is more effective than a higher dose in modulating social cognition and that nasal valve dimensions (summed minimum cross-sectional area) are associated with treatment response in a treatment condition where brain effects occur. Moreover, these results provide behavioral evidence that OT delivered intranasally using a Breath Powered bi-directional device reaches the brain and influences social cognition, whereas IV administered OT, which similarly increased plasma OT concentration, did not.

These data highlight the subtle effect of OT on the processing of emotionally ambiguous facial stimuli in relation to anger perception, as there was no difference in the ratings of angry or happy faces. Although the specific effects of OT in response to emotionally ambiguous stimuli indicate that OT only influences the emotional assessment of stimuli which are non-abundant with overt cues, the lack of effects in the happy and angry stimuli could also be explained by the relatively low variability in the ratings of these stimuli by healthy volunteers. There were also no differences in ratings of trust for all facial stimuli between treatments, however, there was a main effect for treatment on the ratings of happy faces, reflecting differential ratings of trustworthiness of happy faces between experimental treatments, although pairwise comparisons did not reveal any significant differences after false discovery rate correction. Although the reported effects of OT increasing in-group trustworthiness within economic paradigms appear robust,^6, 8^ observed effects on rating trustworthiness in faces seem to be smaller.^64^ Perceived trustworthiness in faces may produce smaller effects or not be as sensitive as other measures of trust. The data also indicate that peripherally administered OT increases blood cortisol concentrations compared with placebo and intranasal OT. Although there was no significant interaction between treatment and time, this provides preliminary evidence that peripherally administered OT may increase hypothalamus–pituitary–adrenal axis activation but intranasally administered OT has no such effects.

Converging biological and behavioral evidence suggests that lower OT doses may be more efficacious than higher doses. For instance, compared with higher doses, lower doses increased peripheral levels of OT in saliva,^65^ attenuated cortisol stress responses^66^ and increased eye gaze in patients with Fragile X syndrome.^67^ In animals, a low dose of OT administered shortly after birth increased partner preference later in life, whereas higher doses did not.^68^ Similarly, lower doses have been associated with stronger increases in social recognition compared with higher doses.^69, 70^ The dose–response data reported here provide useful preliminary evidence concerning the optimal dose for social cognition modulation; however, extrapolation from healthy individuals to patients must be with caution. Patients with social-cognitive deficits may respond differently than healthy volunteers, so future studies should explore effects in patient populations to determine the generalizability of these findings to target illnesses. Future work should also further investigate the role of different delivery devices, administration routes, dosages and social cognition tasks on the efficacy of intranasal OT, ideally using larger sample sizes given the limitation of a relatively small sample size in the present study.

The nasal valve is the point of greatest resistance for airflow in the nasal cavity. We show that participants with larger nasal valve dimension rate ambiguous faces as less angry, suggesting that nasal cavity anatomy has a role in determining the social-behavioral response to intranasal OT administration. This role of nasal anatomy emphasizes the importance of the method of nasal delivery, and is consistent with the idea that deposition pattern and nose-to-brain activity influence treatment effect. Despite the increasing cost of psychiatric illness,^71^ the development of new therapeutics has slowed dramatically.^72, 73^ Although the development of novel molecules is certainly important, innovation in the method of intranasal administration may breathe new life into the use of OT—which has already shown promise for the treatment of psychiatric illness^12, 13^—by increasing CNS activity, therapeutic index and reliability of action by directing delivery more effectively to upper posterior target areas and by addressing barriers related to the nasal anatomy.

There are a number of interpretations regarding why no effect was observed at the 24 IU OPN-OT dose, in contrast to the 8 IU dose. For example, a higher OT dose is more likely to influence the balance of AVP/OT, as evidenced by the decrease in AVP concentration after 24 IU OPN-OT (but not 8 IU OPN-OT) observed in the present study, which can modulate social behavior.^49^ Much like OT, AVP receptors are located both centrally and peripherally^74, 75^ and have an important role in social behavior and psychopathology.^49^ However, further investigation is required as these results are in contrast with past research measuring AVP in saliva after OT administration.^76^ Relatedly, observed levels of plasma OT just after the completion of the social cognition task were noticeably lower than past research,^35, 77, 78^ suggesting targeted OT delivery using the Breath Powered device may reduce systemic exposure while still producing central effects.^79, 80^ It is worth noting, however, that the use of the Breath Powered device in the current study renders direct comparison with these past studies difficult. Moreover, given the range of administration techniques used in OT trials, and the high variability known to exist in drug deposition patterns with nasal delivery devices, there likely already exists a degree of variability in OT bioavailability within and between studies.

A growing body of evidence suggests that OT modulates social cognition in humans, particularly negatively valenced emotions.^81^ The present data are largely consistent with results from past studies in that differences were only discovered on the perception of anger in emotionally ambiguous faces. Prior studies suggest that OT reduces bias towards negative information in anxious individuals^82, 83, 84^ and decreases aversion to angry faces in healthy adults,^44^ however, this is the first study to the authors' knowledge to report data suggesting a reduction of perceived negativity in healthy individuals. Such results have important implications for disorders that are characterized by a negative bias towards social stimuli (for example, social anxiety disorder).

In summary, our study presents new insights in relation to an improved method of targeted intranasal OT delivery, and shows a specific social-cognitive response after using the Breath Powered device for delivery of OT compared with IV delivery producing similar systemic exposure, suggesting that direct nose-to-brain activity is being achieved. In addition, this study provides preliminary evidence that a lower dose (8 IU) may offer greater efficacy than a higher dose (24 IU) when administered with the Breath Powered device.

## Figures and Tables

**Table 1:** Participant ratings in the social cognition task

*Outcomes*	*8* *IU OPN-OT*	*24* *IU OPN-OT*	*IV OT*	*Placebo*	*Linear mixed-model main effect*		
					*df*	*F*	P
*Emotional expression evaluation*							
Angry ratings of ambiguous faces	2.11 (0.15)	2.46 (0.17)	2.32 (0.18)	2.41 (0.15)	3,14.72	7.62	0.003
Happy ratings of ambiguous faces	2.61 (0.14)	2.67 (0.12)	2.38 (0.14)	2.51 (0.13)	3,15.17	1.78	0.193
Angry ratings of angry faces	3.51 (0.2)	3.54 (0.16)	3.68 (0.2)	3.57 (0.16)	3,14.76	0.82	0.505
Happy ratings of angry faces	4.15 (0.62)	4.26 (0.57)	4.29 (0.54)	4.3 (0.36)	3,15	0.32	0.314
Angry ratings of happy faces	1.23 (0.02)	1.25 (0.02)	1.24 (0.02)	1.24 (0.02)	3,15	0.97	0.433
Happy ratings of happy faces	4.11 (0.16)	4.26 (0.14)	4.31 (0.13)	4.3 (0.09)	3,13.84	1.32	0.309
							
Trustworthiness	3.13 (0.04)	3.15 (0.05)	3.16 (0.05)	3.11 (0.03)	3,14.27	2.57	0.095

Abbreviations: IU, international unit; IV, intravenous; OPN-OT, OT delivered with the Breath Powered OptiNose device; OT, oxytocin.

Unless specified otherwise, values are estimated means based on linear mixed models with standard error in parenthesis.

## Notes

PGD is an employee of OptiNose AS, Oslo, Norway and owns stock and stock options in OptiNose. RAM is an employee of OptiNose US, Yardley, PA, USA and owns stock and stock options in OptiNose. OAA has received speaker's honoraria from GSK, Lundbeck and Otsuka for work not directly relevant to the submitted manuscript. MT has received speaker's honorarium from Medivir for work not directly relevant to the submitted manuscript. MR and KTS are employed (and KTS owner) by Smerud Medical Research International AS, a CRO receiving fees for clinical trial services from OptiNose AS. The remaining authors declare no conflict of interest.
