# Supplementary material for: Low-dose oxytocin delivered intranasally with Breath Powered device affects social-cognitive behavior: a randomized four-way crossover trial with nasal cavity dimension assessment
Source: Transl Psychiatry. 2015 Jul 14;5(7):e602–. doi: 10.1038/tp.2015.93 (PMC5068727; doi:10.1038/tp.2015.93)
Supplement: Supplementary Information [file tp201593x1.doc]

**Low dose oxytocin delivered intranasally with Breath Powered device affects social-cognitive behavior: a randomized 4-way crossover trial with nasal cavity dimension assessment**

***Supplementary Information***

**1) Pilot study**

An open label pilot study comparing the pharmacokinetics of 24IU OPN-OT and 1IU IV-OT was conducted with 5 eligible participants (4 of these participants participated in the main study) to ensure that 1IU IV-OT would equal or exceed the serum exposure following IN-OT treatment. The IV OT (1IU) was mixed in 200ml 0.9% NaCl and administered at an infusion rate of 600ml/hour over 20 minutes. Blood was sampled just before administration (baseline) and at seven timepoints after completion of administration (10, 20, 30, 45, 60, 90, and 120 minutes). The pilot data indicated that peripheral (serum) OT levels following 1IU IV OT reasonably approximated the levels achieved after 24IU OPN-OT administration during important timepoints post-administration known to correspond changes in behavior (i.e., 30 to 60 minutes; see supplementary figure S1). There was a one-month washout period, at the minimum, between participation in the pilot study and the experimental phase.

**2) Intranasal spray ingredients and administration regime**

Each OT bottle contained 40IU of OT per ml, with each spray providing a 4IU dose. Each ml of solution contained 0.2mg of propyl parahydroxybenzoate and 0.4 mg of methyl parahydroxybenzoate. Other excipients included chlorobutanol, disodium phosphate anhydrous, citric acid anhydrous, sodium chloride, glycerol, sorbitol solution 70%, and purified water. The placebo intranasal formulation, which contained all excipients expect the active ingredient.

To ensure the same volume was dispensed into the nasal cavity, three puffs were administered per nostril (alternating between each nostril), regardless of treatment arm. For each treatment arm, the first two puffs were self-administered from bottle “A2”, and the next four puffs were self-administered from bottle “B4”, respectively. Depending on the randomization sequence, participants received either 8IU OPN-OT [two, 4IU OT puffs in each nostril from bottle A2 and four placebo puffs (two in each nostril) from bottle B4] with placebo IV (treatment A), 24IU OPN-OT [two, 4IU OT puffs in each nostril from bottle A2 and four, 4IU OT puffs (two in each nostril) from bottle B4] with placebo IV (treatment B), placebo nasal treatment [OPN-PBO; two placebo puffs in each nostril from bottle A2 and four placebo puffs (two in each nostril) from bottle B4] with 1IU OT IV (treatment C), or OPN-PBO [two, placebo OT puffs in each nostril from bottle A2 and four placebo puffs (two in each nostril) from bottle B4] with placebo IV (condition D).


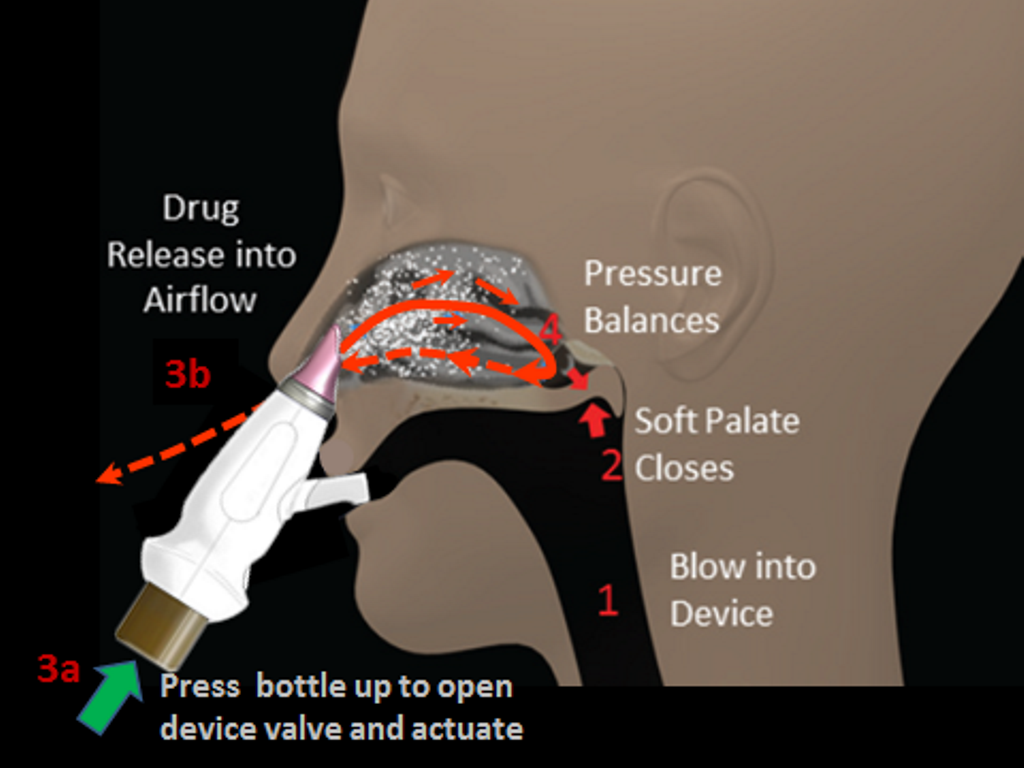


**Figure S1. The Breath Powered, closed-palate, bi-directional nasal delivery device.** The user slides the device into the nostril and then blows into the device (1). The intraoral pressure created by blowing into the mouthpiece closes the soft palate and creates an airtight seal (2), isolating the nasal cavity from the rest of the respiratory system. Shortly after blowing into the device, the user actuates the spray pump (3a), which releases the pressure of the user’s exhaled breath and propels the drug to targeted nose-to-brain regions in the upper posterior nasal cavity (3b). The airflow balances pressure across the soft palate (4) enabling the exhaled breath to travel the in the opposite direction, exiting out the opposite nostril.

**Figure S2. Pilot study assessing the pharmacokinetics of plasma OT after the administration of 24IU IN-OT and IV-OT.** The social cognitive task was not performed during the pilot study thus the ‘Social cognition task’ period shown is purely illustrative of when this would occur. Error bars represent standard error of the mean.

**
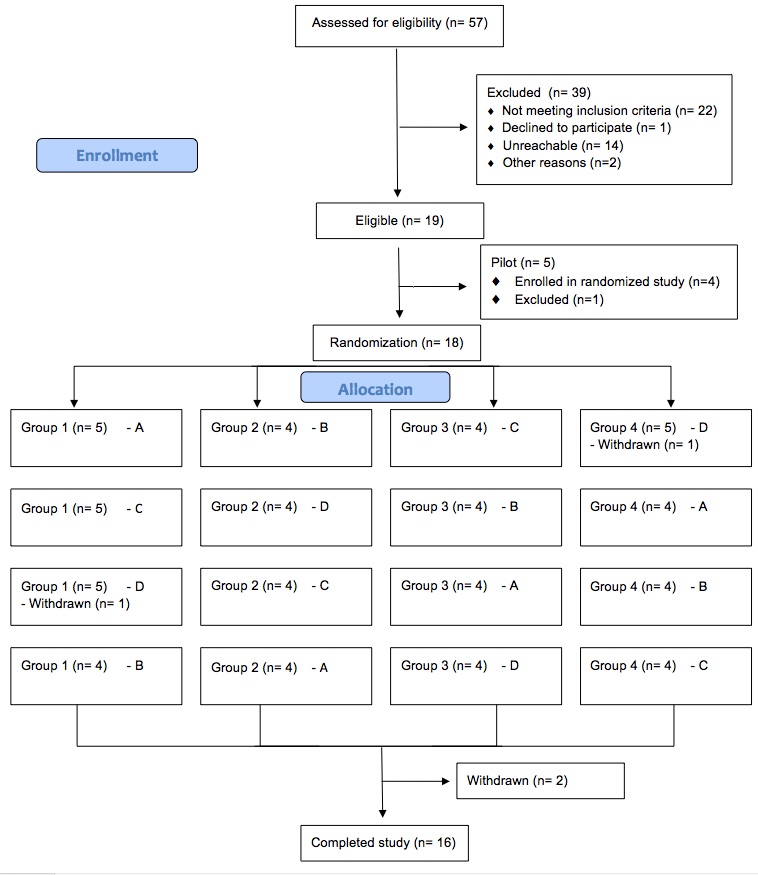
**

**Figure S3. CONSORT diagram.**

**Figure S4. Percentage change of plasma oxytocin after the administration of 8IU OPT-OT, 24IU OPT-OT, IV-OT, and placebo.** Error bars represent standard error of the mean.

**Figure S5. Pharmacokinetics of plasma cortisol after the administration of 8IU OPT-OT, 24IU OPT-OT, IV-OT, and placebo.** Error bars represent standard error of the mean.
